# Supplementary material for: Dynamic Changes in the Gut Microbial Community and Function during Broiler Growth
Source: Microbiol Spectr. 2022 Aug 11;10(4):e01005-22. doi: 10.1128/spectrum.01005-22 (PMC9430649; doi:10.1128/spectrum.01005-22)
Supplement: Supplemental file 1 — Supplemental material. Download spectrum.01005-22-s0001.pdf, PDF file, 0.6 MB [file spectrum.01005-22-s0001.pdf]

SUPPLEMENTARY FIGURES

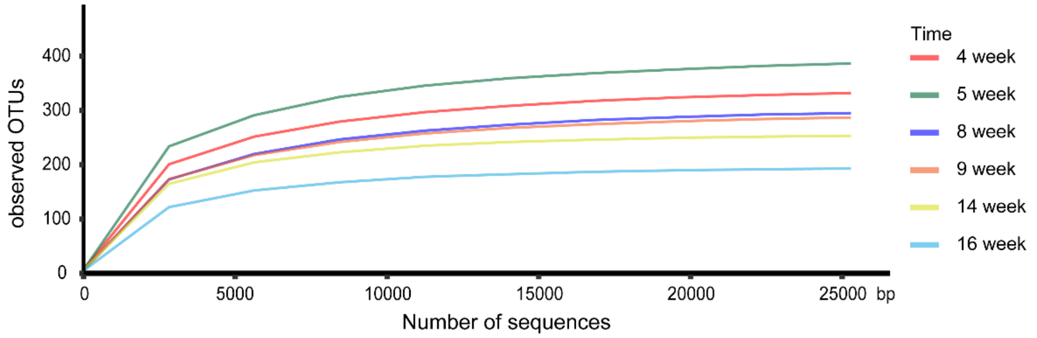

Figure S1. Relationship between sequence number and detected OTUs (rarefaction curves).

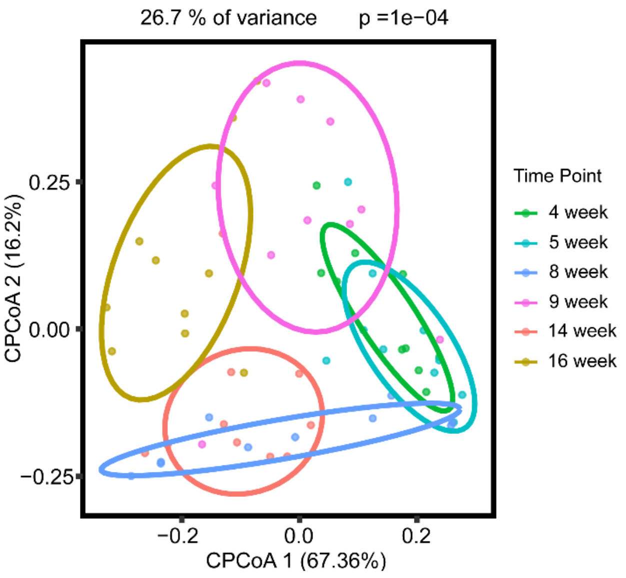

Figure S2. Constrained PCoA plot of Bray Curtis distances constrained by sampling time points.

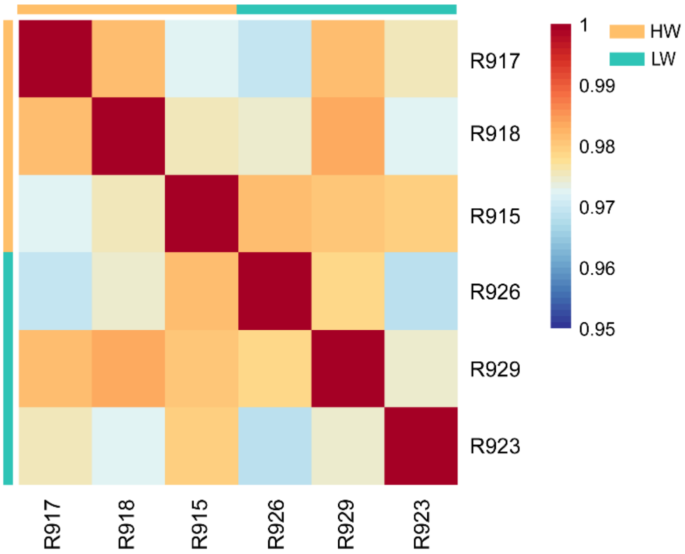

Figure S3. Spearman correlation heatmap of transcriptome samples. Blue and red represent low and high correlation, respectively. R92_X represents the LW group and R91_X represents the HW.

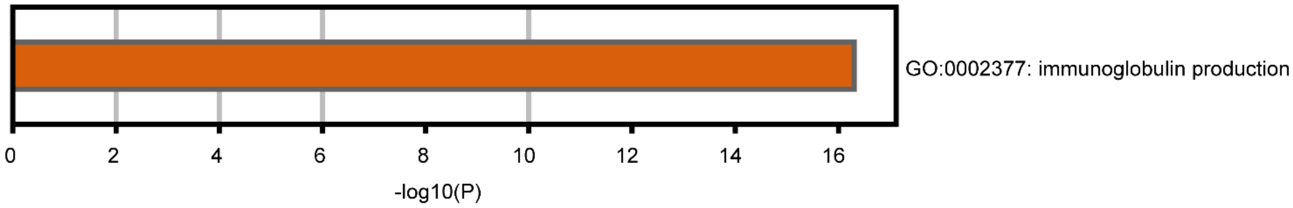

Figure S4. Go term enriched by differentially expressed genes.

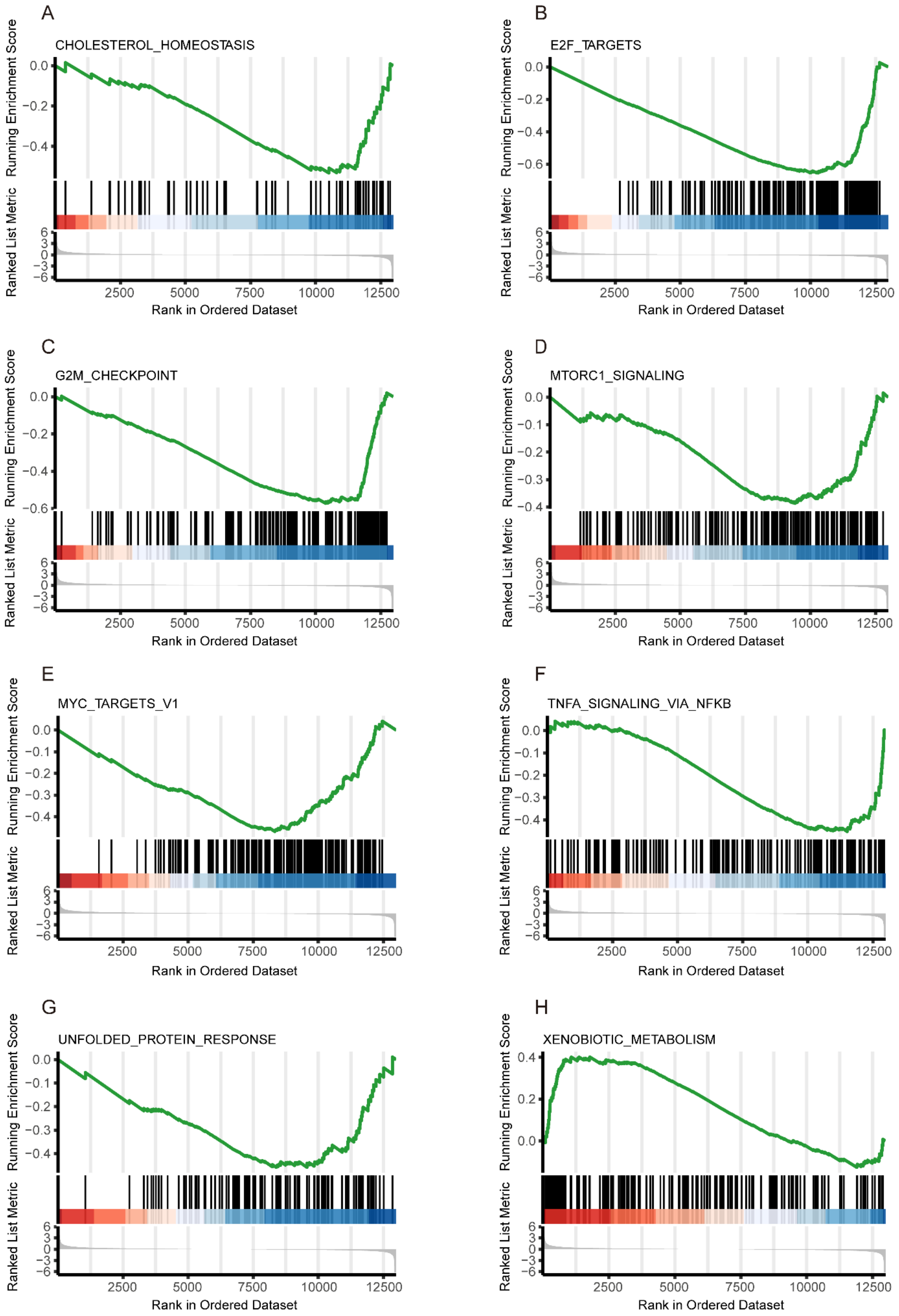

Figure S5. 8 significant pathways in GSEA analysis (FDR, *P*adj < 0.05).

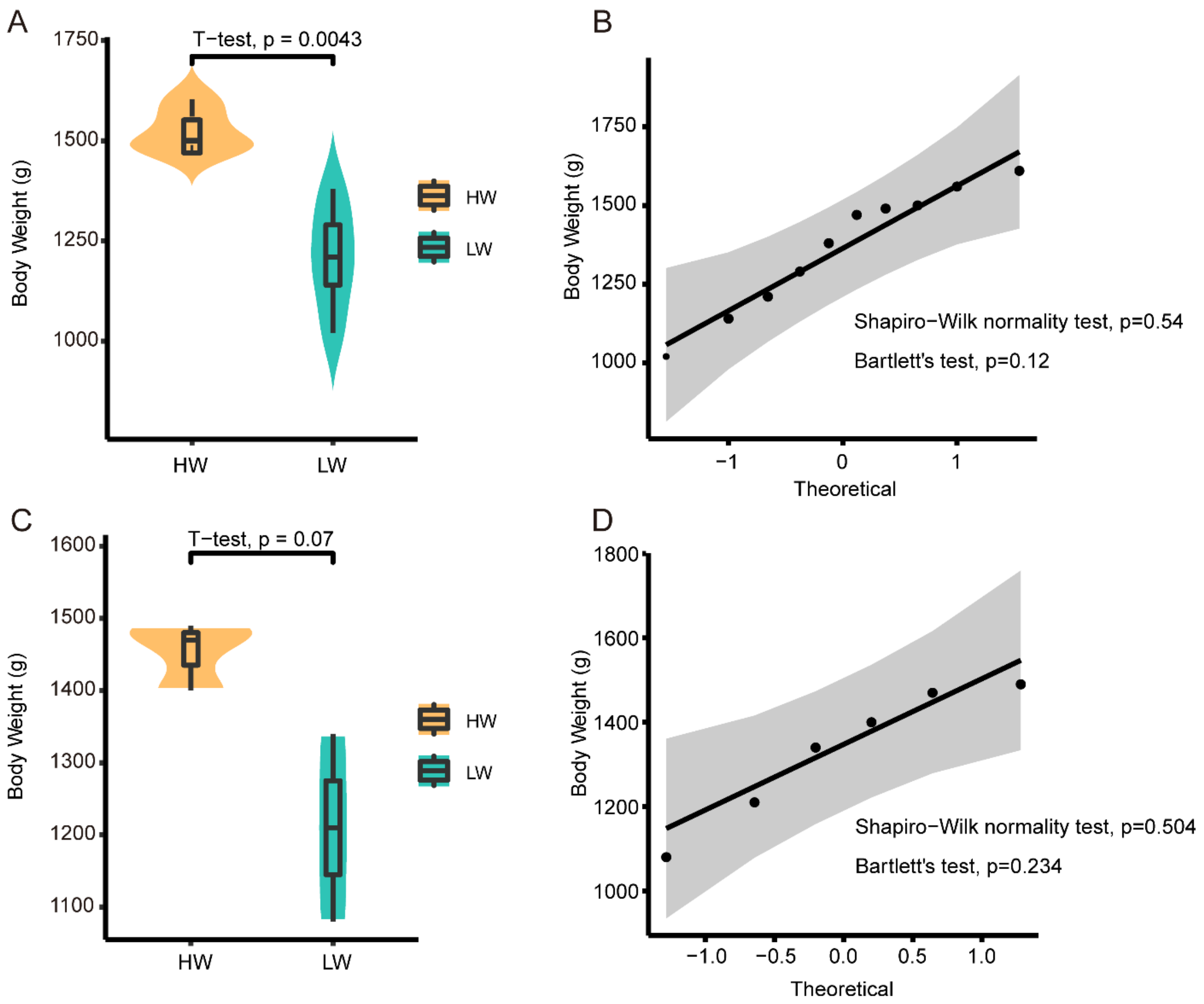

Figure S6. HW and LW grouping rationality test. (**A**) Comparison of body weight difference between HW and LW of 16S rRNA gene sequencing samples in week 9. (**B**) 16S rRNA gene sequencing samples weight distribution QQ-plot in week 9. Shapiro−Wilk normality test for distribution normality test. Bartlett's test for variance consistency test. (**C**) Comparison of body weight difference between HW and LW of transcriptome sequencing samples in week 9. (**D**) Transcriptome sequencing samples weight distribution QQ-plot in week 9. Shapiro−Wilk normality test for distribution normality test. Bartlett's test for variance consistency test.
